# The Ergogenic Effects of Acute Carbohydrate Feeding on Resistance Exercise Performance: A Systematic Review and Meta-analysis

**DOI:** 10.1007/s40279-022-01716-w

**Published:** 2022-07-09

**Authors:** Andrew King, Eric Helms, Caryn Zinn, Ivan Jukic

**Affiliations:** 1 Sport Performance Research Institute New Zealand (SPRINZ), Auckland University of Technology, 17 Antares Place, Mairangi Bay, Auckland, 0632 New Zealand; 2 School of Engineering, Computer and Mathematical Sciences, Auckland University of Technology, Auckland, New Zealand

## Abstract

**Background:**

Carbohydrate (CHO) ingestion has an ergogenic effect on endurance training performance. Less is known about the effect of acute CHO ingestion on resistance training (RT) performance and equivocal results are reported in the literature.

**Objective:**

The current systematic review and meta-analysis sought to determine if and to what degree CHO ingestion influences RT performance.

**Methods:**

PubMed, MEDLINE, SportDiscus, Scopus, and CINAHL databases were searched for peer-reviewed articles written in English that used a cross-over design to assess the acute effect of CHO ingestion on RT performance outcomes (e.g., muscle strength, power, and endurance) in healthy human participants compared to a placebo or water-only conditions. The Cochrane Collaboration’s risk of bias tool and GRADE approaches were used to assess risk of bias and certainty of evidence, respectively. Random effects meta-analyses were performed for total training session volume and post-exercise blood lactate and glucose. Sub-group meta-analysis and meta-regression were performed for categorical (session and fast durations) and continuous (total number of maximal effort sets, load used, and CHO dose) covariates, respectively.

**Results:**

Twenty-one studies met the inclusion criteria (*n* = 226 participants). Pooled results revealed a significant benefit of CHO ingestion in comparison to a placebo or control for total session training volume (standardised mean difference [SMD] = 0.61). Sub-group analysis revealed a significant benefit of CHO ingestion during sessions longer than 45 min (SMD = 1.02) and after a fast duration of 8 h or longer (SMD = 0.39). Pooled results revealed elevated post-exercise blood lactate (SMD = 0.58) and blood glucose (SMD = 2.36) with CHO ingestion. Meta-regression indicated that the number of maximal effort sets, but not CHO dose or load used, moderates the effect of CHO ingestion on RT performance (beta co-efficient [*b*] = 0.11). Carbohydrate dose does not moderate post-exercise lactate accumulation nor do maximal effort sets completed, load used, and CHO dose moderate the effect of CHO ingestion on post-exercise blood glucose.

**Conclusions:**

Carbohydrate ingestion has an ergogenic effect on RT performance by enhancing volume performance, which is more likely to occur when sessions exceed 45 min and where the fast duration is ≥ 8 h. Further, the effect is moderated by the number of maximal effort sets completed, but not the load used or CHO dose. Post-exercise blood lactate is elevated following CHO ingestion but may come at the expense of an extended time-course of recovery due to the additional training volume performed. Post-exercise blood glucose is elevated when CHO is ingested during RT, but it is presently unclear if it has an impact on RT performance.

**Protocol Registration:**

The original protocol was prospectively registered on the Open Science Framework (Project identifier: 10.17605/OSF.IO/HJFBW).

**Supplementary Information:**

The online version contains supplementary material available at 10.1007/s40279-022-01716-w.

## Key Points

**Table:** 

Results of the current meta-analysis indicate that carbohydrate ingestion before and during resistance training allows for greater volume to be completed during sessions lasting longer than 45 min and consisting of at least 8–10 sets.
The ingestion of carbohydrate after a fast of 8 h or more, such as the overnight fast, can be expected to improve resistance training performance.
Post-exercise blood lactate is elevated with carbohydrate ingestion, likely due to the additional volume of work completed. Therefore, a trade-off may exist where the cost of the ergogenic effect of carbohydrate ingestion on RT volume induces additional fatigue and could influence time-course of recovery. Post-exercise blood glucose was elevated with carbohydrate ingestion, where readily digestible sources ingested during training seem to increase blood glucose the most.

## Introduction

Dietary carbohydrate (CHO) and fat are the two main fuel sources during exercise, but the relative contribution of each depends on the intensity and duration of exercise [1], with CHO making a greater relative contribution to energy production where exercise is of moderate-to-high intensity [2]. Dietary CHO is stored in the liver and skeletal muscle as glycogen and is generally considered important for fuelling high-intensity exercise [3]. Resistance training (RT) is often performed intermittently and at high intensity by athletes seeking strength, power, and hypertrophy adaptations [4]. While the role of CHO in endurance exercise performance has received thorough study with general recommendations for ingestion in the pre-, intra-, and post-exercise periods [5–7], the role of CHO feeding on RT performance is less clear due to conflicting findings in a relatively smaller body of literature [8]. The stressors and energetic demands of RT differ from endurance training [9, 10], and given the high-intensity nature of RT, CHO ingestion needs to be considered with specificity to the unique stimuli and demands of RT.

Standard volumes of RT result in decreases of total muscle glycogen stores of 24– 40% [11–14], with greater training volumes resulting in greater decrements [15]. Muscle glycogen is compartmentalised to several distinct locations within skeletal muscle, including stores that are intra-myofibrillar (i.e., within the muscle fibre), intermyofibrillar (i.e., between muscle fibres), and subsarcolemmal (i.e., between the outermost myofibers and the sarcolemma) [16]. While the exact metabolic role of these muscle glycogen compartments requires further elucidation, intra-myofibrillar stores of glycogen are purported to be located such that they are readily available to fuel Ca^2+^ release from the sarcoplasmic reticula [17–19]. Recently, Hokken et al. [20] reported that in addition to modest decreases in total glycogen stores of the *M. vastus lateralis* (38%) after a lower body RT session, approximately half of type II fibres exhibited near total depletion of intra-myofibrillar stores of glycogen. Thus, the reductions in total muscle glycogen and the selective depletion of intra-myofibrillar glycogen incurred during RT could impair the contractile ability of muscle and play a role in fatigue.

Glycogenolysis occurs during strenuous exercise such as RT, but also during periods of fasting, such as the overnight fast. Overnight fasting significantly decreases hepatic stores of glycogen but has a negligible effect on muscle glycogen [21–23]. Despite the minimal effect of overnight fasting on muscle glycogen stores, the ingestion of a mixed, medium–high CHO meal after an overnight fast in rested individuals increases muscle glycogen stores by 12–42% [24–27]. Thus, the duration of fast before RT likely influences CHO availability during training, which could be attenuated with CHO ingestion. In addition to off-setting exercise- and fasting-induced decrements to glycogen content, CHO feeding may also enhance RT performance by maintaining/increasing blood glucose concentration as a readily available fuel source [28, 29], or by activating oropharyngeal receptors sensitive to CHO presence that relay signals to regions of the brain involved in motivation, reward, and motor output [30, 31]. Taken together, several metabolic and central mechanisms related to CHO feeding could potentially improve RT performance.

The literature investigating the effects of acute CHO feeding on RT performance is equivocal. Carbohydrate ingestion does not seem to enhance peak power [32], maximal strength [33], or peak isokinetic force or torque [34–37]. However, CHO ingestion often improves RT performance indices such as total isokinetic work completed [35] and total number of sets and repetitions completed to failure [38, 39], especially during longer (> 45 min) RT sessions [32, 35, 38, 39]. With that said, not all studies agree, as some have reported null findings during longer RT protocols with CHO ingestion for similar RT performance indices such as total repetitions to failure [40, 41]. Likewise, for shorter duration training sessions (< 45 min), no ergogenic effect of CHO ingestion was reported on lower body sets or repetitions to failure [42, 43], nor was CHO supplementation reported to improve lower and/or upper body isokinetic total and average work in pre- versus post-RT session comparisons [34, 37]. But once again, exceptions exist as some shorter duration studies do report an ergogenic effect of CHO ingestion on RT performance [44–46]. Thus, while there seems to be a general trend for CHO ergogenicity that is dependent on RT session duration, these findings are not consistent and are at times contradictory. Recently, a systematic review by Henselmans et al. [47] concluded that while the majority of studies investigating the acute effects of CHO ingestion on RT performance did not find a positive effect, there was a trend where studies with longer pre-exercise fast durations and RT protocols of greater than 10 sets completed reported an ergogenic effect of CHO ingestion. However, it is important to note that a quantitative analysis of specific RT outcomes was not conducted.

These inconsistencies and gaps in the literature establish a need for a comprehensive review and quantitative synthesis of the available literature on CHO ingestion’s effect on RT performance. Thus, we conducted a systematic review and meta-analysis on the effects of acute CHO ingestion on RT performance to understand if and to what degree carbohydrate feeding influences RT performance, assess the certainty of evidence presented in the literature, and identify gaps in knowledge for future investigations. Such evidence is necessary to guide RT fuelling recommendations for athletes, coaches, and nutrition practitioners.

## Methods

### Registration of Systematic Review Protocol

A systematic review was performed in accordance with the *Cochrane Handbook for Systematic Reviews* (version 5.1.0) and the Preferred Reporting Items for Systematic Reviews and Meta-Analyses (PRISMA) [48]. The original protocol was prospectively registered on the Open Science Framework (Project identifier: 10.17605/OSF.IO/HJFBW). The protocol registration occurred after pilot searches but before any formal systematic searches were conducted.

### Literature Search

A patient/population, intervention, comparison, and outcomes (PICO) strategy was developed using the Word Frequency Analyser Tool (https://sr-accelerator.com/#/help/wordfreq) to suggest search terms for electronic databases. PubMed, MEDLINE, SPORTDiscus, Scopus, and CINAHL electronic databases were searched from inception to 26^th^ of June 2021. The MEDLINE, SportDiscus, and CINAHL strategies were run simultaneously as a multi-file search in EBSCOhost and the records yielded from this search were automatically deduplicated by EBSCOhost. Free-text terms were chosen based on word frequency analysis using the Researcher Refiner tool (https://ielab-sysrev2.uqcloud.net/) and pilot searches to achieve a balance between sensitivity and precision. Only terms related to or describing the intervention were used in the search. The following keywords were used to search the PubMed/MEDLINE database and were applied to the title, abstract, and keyword search fields: “carbohydrate” OR “glucose” OR “maltodextrin” AND “resistance training” OR “resistance exercise” OR “strength training” OR “weight training”. The full search strategy for each respective electronic database is available in the Supplementary Information Appendix S1. Secondary searches included (a) forward citation tracking of included studies using Google Scholar and (b) setting up search alerts of the electronic databases included in this systematic review up to the 8^th^ of January 2022. No year or any other restrictions were applied in the search.

### Text Screening

Search records were imported into Endnote (version X8.2, Clarivate Analytics, Philadelphia, PA, USA) and duplicates were removed using automated and manual methods. The remaining records were uploaded to the systematic review tool Rayyan (https://rayyan.ai/). Records were independently screened by title and abstract by two investigators (AK and IJ) to determine initial eligibility. The full texts of the remaining records were then retrieved and assessed by the same investigators for inclusion in the review. Disagreements between investigator’s decisions were resolved via discussion and consensus or in consultation with a third reviewer (EH) where required.

### Inclusion and Exclusion Criteria

All studies included in this systematic review met the following inclusion criteria: (1) the study was a peer-reviewed research article; (2) was written in the English language; (3) included healthy human participants with no musculoskeletal injury; (4) used a cross-over study design to assess the acute effect of carbohydrate ingestion in the pre- and/or intra-exercise period on outcomes of muscle force production (e.g., maximal strength and power) and/or muscle endurance; and (5) used a low to zero-caloric placebo (≤ 25 total kilocalories) or water only comparator condition. Performance indices considered for inclusion were those related to muscle force production (e.g., 1 repetition-maximum [1-RM], isokinetic/isometric force production, power) and endurance (e.g., repetitions completed per set or exercise, total session work or volume, session duration). Perceptual measures (e.g., perceived exertion) and metabolic markers (e.g., blood lactate and glucose) were considered secondary outcomes of interest. Review articles, unpublished abstracts, theses, and dissertations were excluded.

### Study Coding and Data Extraction

From the included studies, the following data were extracted: (1) study design descriptors including information about blinding and the number of periods and sequences; (2) the number of participants in the study and characteristics such as age, sex, body mass, height, and training experience; (3) pre-trial diet standardisation including length and method of dietary tracking; (4) pre-testing fast duration; (5) the dose, timing, and type of carbohydrate used; (6) description of the comparator placebo and/or control condition/s; (7) the RT protocol including intensity, volume, rest periods, exercise selection, and session duration; and (8) means and standard deviations of the relevant performance, perceptual, and metabolic indices. Means and standard deviations for all primary and secondary outcomes were collated into a single spreadsheet and sorted by outcome. Where insufficient information was reported, the corresponding author of the study was contacted via email. All data extraction was completed independently by two authors (AK and IJ). Coding files were cross-checked between the two authors and differences were resolved via discussion and consensus.

### Risk of Bias

Risk of bias was assessed using the Cochrane Collaboration’s risk of bias tool for randomised trials (RoB 2) [49] with online resources for cross-over trial designs (https://www.riskofbias.info/welcome/rob-2-0-tool/rob-2-for-crossover-trials). Risk of bias was assessed using the information provided in the published article. Rating and grading were completed independently by two investigators (AK and IJ). Decisions were made using the Cochrane Collaboration’s most recent online guiding document for cross-over trial designs (March 2021). Risk of bias related to blinding was considered important in this review since risk of bias is highest when affected by subjective expectations and that blinding would be conceivably easy to apply [50]. Signalling question 4.2 of the guidance document was adjusted to consider the risk of bias arising from diet standardisation and the time of day at which trials were conducted. Inconsistent diet standardisation could affect CHO availability before the RT testing protocol which could influence performance [51], and exercise performance is known to be affected by the time of day at which it is performed [52]. Differences in risk of bias assessment were resolved via discussion and agreement before merging the scores into a single spreadsheet.

The Grading of Recommendations Assessment, Development and Evaluation (GRADE) system was used to evaluate the certainty of evidence for the studies included in the quantitative synthesis [53], in a similar manner to previous reviews evaluating exercise physiology and performance outcomes [54–56]. Specifically, a study was rated high and downgraded one point to moderate, low, or very low for each of the following limitations: imprecision, inconsistency, and risk of bias. For imprecision, a study was downgraded if the conclusion about the effect magnitude (i.e., point estimate) would be altered based on the lower or upper boundary of the confidence interval (CI). For example, if the mean effect was moderate and the lower bound of the 95% CI crossed the threshold for a small effect size (i.e., g < 0.5), the precision was insufficient to support a strong recommendation of the conclusion because the lower bound of the CI could include a small effect. For inconsistency, a study was downgraded if high statistical heterogeneity was observed (*I*^2^ > 50%), and for risk of bias if > 50% of the studies had > 1 risk of bias item assessed as high risk.

### Statistical Analysis

A random-effects meta-analysis was performed for each separate outcome when reported by at least two studies in the review. Meta-analysis was performed in R language and environment for statistical computing (version 4.0.5, The R foundation for Statistical Computing, Vienna, Austria) [57], using the *Meta* and *Metafor* statistical packages [58, 59]. The restricted maximum-likelihood method was used to calculate model parameters, and the inverse variance method was used to pool a weighted estimation of the standardised mean differences across the studies included in the quantitative synthesis [60]. The Knapp–Hartung small-sample correction was also used as it provides a more adequate accounting of uncertainty when pooling treatment effects from a small number of heterogeneous studies [61, 62]. Three outcomes of interest were sufficiently reported by the included studies to enable meta-analysis: these were—total training volume, blood lactate, and blood glucose. For total training volume, repetitions completed to failure were most reported and preferentially used in the meta-analysis.

Standardised mean differences (SMD) with Hedge’s g correction and 95% CIs (lower bound, upper bound) were calculated between CHO and placebo/control condition trials using the means and standard deviations of RT performance and metabolic outcomes, the correlation between the trials, and the number of participants [63]. Since no studies reported correlations, corresponding authors were contacted via email to request the data. The requested studies were either too old and the data had been destroyed/lost or no reply was received, so correlations were calculated using unpublished data (*n* = 5) from our laboratory for the outcomes of interest. These calculations yielded values of 0.78, 0.74, and 0.26 for training volume, blood lactate, and blood glucose, respectively. Sensitivity analyses were performed using correlation values of 0.3 and 0.5 for training volume and blood lactate, and 0.5 and 0.7 for blood glucose, to check the robustness of the results. Standardised mean difference magnitude was interpreted as: small (0.20–0.49), moderate (0.50–0.79), and large (> 0.80) [64]. All hypothesis tests were conducted with significance set at *α* = 0.05. The number of studies is denoted by *k*. Where multiple observations of an outcome were reported (e.g., separate effects were reported for repetitions to failure per exercise, rather than total session repetitions completed), the observations were combined into a single, composite effect using methods outlined by Borenstein et al. [63] for dependent continuous outcomes. This ensured that double counting individuals from those studies included in the meta-analysis was avoided.

Meta-regressions based on CHO dose (g/kg body mass), load used (% 1 − RM), and total number of maximal effort sets, were performed when at least six effects were reported for each outcome [65] and are presented as unstandardised regression co-efficient *b*. The statistical heterogeneity of the trials included in the meta-analysis was assessed by the *I*^2^ statistic, where *I*^2^ was considered small (*I*^2^ < 25%), moderate (*I*^2^ = 25–49%), or high (*I*^2^ > 50%) [66]. Publication bias was assessed by examining funnel plot asymmetry and using Egger’s regression test [67] for primary outcomes with more than 10 studies, as recommended in the *Cochrane Handbook for Systematic Review Interventions* [68]. Additional information concerning (a) decisions on indices to be included in the total session volume meta-analysis, (b) composite effect calculations, (c) decisions on meta-regression calculations, and (d) decisions on publication bias analysis are detailed in Supplementary Information Appendix S2.

## Results

### Search Results

**Fig. 1:**
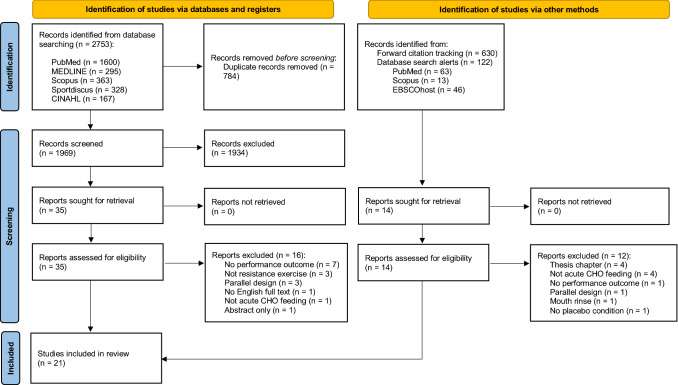
Literature search flow chart. *CHO* carbohydrate, *n* number of studies,

The initial search yielded 2753 records, of which 1969 were screened by title and abstract after duplicates were removed. Title and abstract screening yielded 35 potential inclusions that were screened by full text, and 19 of these studies met the full inclusion criteria. Monitoring newly published articles with search alerts did not yield any additional inclusions. Forward citation tracking yielded two additional studies that met the inclusion criteria, resulting in 21 studies included in this review. The stages of this search and the study selection process are presented in Fig. 1.

### Study Characteristics

#### Participants

There were 232 participants pooled across all studies in this review. However, two studies [44, 45] used the same participant data for the analysis, reducing the total participants to 226. Of the 226 pooled total participants, 214 were male and 12 were female. Of the 21 studies in this review, 19 included a male-only sample, one study included only females [33], and one study recruited a mixed sex cohort [36]. All studies were conducted in young adult populations, with the mean age between 20 and 30 years. Participants in 16 studies were described as resistance trained or as athletes in sports involving resistance training. There was a range of RT experience with some studies requiring a minimum 2–6 months of training experience [41, 42, 69]; whereas others reported participant cohorts with more than 5 years RT experience [34, 35, 38, 44, 45]. Participants in four studies were recreationally trained or physically active [33, 36, 70, 71] and one study did not report any information regarding training history [72]. A comprehensive description of participant characteristics can be found in Table 1.

**Table 1:** Participant characteristics of individual study samples

Study (year)	Participants	Sex: M/F	Age (years)	Mass (kg)	Training history (subjective description, RT experience (years), relative strength (1RM/BM)
Aoki et al. [33]	CHO = 6; PLA = 6	0/6	22.4 ± 3.8	64.9 ± 7.2	Physically active; at least 2 years; unclear
Ballard et al. [71]	CHO = 21; PLA = 21	21/0	20 ± 1.8	82.3 ± 13.6	Recreationally trained; unclear; unclear
Battazza et al. [72]	CHO = 20; PLA = 20	20/0	25.1 ± 4.4	76.3 ± 7.6	Unclear; unclear; unclear
Bin Naharudin et al. [74]	CHO = 16; PLA = 16	16/0	23 ± 4	77.56 ± 7.13	Resistance trained; at least 2 years; unclear
Bird et al. [73]	CHO = 15; PLA = 15	15/0	21.7 ± 0.8	85.7 ± 1.9	Resistance trained field and court athletes; 3.1 ± 0.3; back squat = 1.55, bench press = 1.1
dos Santos et al. [70]	CHO = 8; PLA = 8	8/0	21.3 ± 2.7	73.1 ± 6.1	Recreationally trained; at least 1 year; relative bench press 1RM = 0.91
Fairchild et al. [36]	CHO = 17; PLA = 17	11/6	22.1 ± 3.9	69.5 ± 9.6	Recreationally active; at least 0.5 years; unclear
Haff et al. [38]	CHO = 6; PLA = 6	6/0	24.3 ± 2.1	82.6 ± 2.6	Resistance trained; 6.2 ± 0.4 years; able to squat 1.5 × BM
Haff et al. [34]	CHO = 8; PLA = 8	8/0	24.3 ± 1.1	85.7 ± 3.5	Resistance trained; 9.9 ± 2.0 years; able to squat 1.75 × BM
Haff et al. [35]	CHO = 8; PLA = 8	8/0	23.7 ± 1.3	94.9 ± 4.9	Resistance trained; 8.1 ± 0.9; unclear
Krings et al. [40]	CHO = 7; PLA = 7	7/0	21.9 ± 1.6	91.6 ± 9.7	Resistance trained; at least 1 year; unclear
Kulik et al. [42]	CHO = 8; PLA = 8	8/0	23.8 ± 1.8	92.9 ± 11.4	Resistance trained; at least 0.5 years; back squat = 1.8 ± 0.2
Lambert et al. [39]	CHO = 7; PLA = 7	7/0	22.8 ± 1.3	82.8 ± 7.7	Resistance trained; at least 2 years; unclear
Laurenson et al. [32]	CHO = 10; PLA = 10	10/0	25.3 ± 6.1	83.6 ± 13.1	Resistance trained; unclear; back squat = 1.63, bench press = 1.28
Naharudin et al. [75]	CHO = 22; PLA = 22; CON = 22	22/0	23 ± 3	77.9 ± 8.1	Resistance trained; 4.7 ± 1.5 years; unclear
Oliver et al. [46]	CHO = 16; PLA = 16	16/0	23 ± 3	88.2 ± 8.6	Resistance trained; at least 2 years; able to squat 1.5 × BM
Rountree et al. [69]	CHO = 8; PLA = 8	8/0	22 ± 1.8	81.3 ± 7.2	CrossFit athletes; at least 0.5 years; unclear
Smith et al. [41]	CHO = 13; PLA = 13	13/0	23 ± 3.8	82.1 ± 11	Resistance trained; at least 2 months; bench press = 1.4 ± 0.2
Wax et al. [44, 45]	CHO = 6; PLA = 6	6/0	29.1 ± 4.4	102.4 ± 20.6	Elite competitive bodybuilders and powerlifters; at least 5 years; unclear
Wilburn et al. [43]	CHO = 10; PLA = 10	10/0	21.6 ± 2.27	90 ± 18.2	Resistance trained; at least 1 year; leg press = 5.98 ± 1.55

Values are expressed as mean ± standard deviation

*BM* body mass, *CHO* carbohydrate, *CON* control, *F* female, *IRM* 1-repetition maximum, *M* male, *PLA* placebo, *RT* resistance training

#### Resistance Exercise Protocol

An exercise protocol including free-weight, isotonic resistance exercise was used in 16 studies, with 7 studies including only lower body exercises [33, 34, 38, 39, 43, 46, 73], one study using upper body only [70], and 7 studies using the upper and lower body [32, 40, 41, 69, 71, 74, 75]. The most common exercise was the back squat, which was used in 7 studies [32, 34, 38, 42, 46, 73, 74], followed by a barbell/dumbbell chest press in 6 studies [32, 40, 41, 70, 71, 74], and leg press in 5 studies [33, 39, 43, 71, 73]. There was a variety of loading schemes from 10 to 100% of 1 − RM, and the total number of sets (including submaximal) completed per session ranged from 3 to 34. Four studies included isokinetic exercise, of which one was knee extension [36] and three were knee extension and flexion [34, 35, 72]. One study [34] included isokinetic contractions in addition to a lower body free-weight RT session. Two studies [44, 45] used a static isometric quadriceps contraction with intermittent bouts of superimposed electrical stimulus. A comprehensive description of each resistance exercise protocol is shown in Table 2.

**Table 2:** Resistance training protocol characteristics of the studies included in the systematic review

Study (year)	Exercises	Exercise protocol (sets x repetitions x load, rest)	Duration (mins)	Outcomes
Aoki et al. [33]	Leg press	1 × 1 × 100% 1RM; 2 × failure × 70%1RM, 1.5-min inter-set	Unclear	1RM load lifted vs pre-exercise Repetitions completed per set
Ballard et al. [71]	Mix of upper and lower body strength/hypertrophy exercises	3 × 10 × 70% 1RM; 1 × failure × 55%1RM, 2-min inter-set and 3-min inter-exercise	80	Volume load per exercise Total session volume load
Battazza et al. [72]	Isokinetic knee extension/flexion	10 × 8 x maximal effort, unclear	29	Pre- vs. post-exercise isometric peak torque Rate of torque development
Bin Naharudin et al. [74]	Back squat, bench press	4 × failure × 90% 10RM, 3-min inter-set	Unclear	Total repetitions completed per set and exercise
Bird et al. [73]	Mix of lower body strength/hypertrophy exercises	4 × failure × 8–15RM, 1.5-min inter-set and 3-min inter-exercise	Unclear	Total session volume CMJ lower body peak power
dos Santos et al. [70]	Bench press	1 × failure × 70% 1RM, unclear	Unclear	Repetitions completed
Fairchild et al. [36]	Isokinetic leg extension	8 × 3 x maximal effort, 5–15-min inter-set	90	Peak and mean repetition force
Haff et al. [38]	Back squat	Failure × 10 × 55% 1RM, 3-min inter-set	CHO: 77.7 ± 19.4 PLA: 46.1 ± 8.9	Total session repetitions and sets Training session duration
Haff et al. [34]	Back squat, speed squat, 1-legged squat	3 × 10 × 10–65% 1RM, 3-min inter-set	38.9 ± 0.3	Total and average isokinetic work (pre- vs post-RT) Total and average torque (pre- vs post-RT)
Haff et al. [35]	Isokinetic knee flexion/extension	16 × 10 x maximal effort, 3-min inter-set	CHO: 56.9 ± 0.2 PLA: 57.1 ± 0.4	Total work per set and session Average and peak torque per set
Krings et al. [40]	Upper and lower body power & strength/hypertrophy exercises	2–6 × 2-failure × 45–90% 1RM, 0.5–3-min inter-set	71.3 ± 2.9	Repetitions to failure in final set of upper body exercises Total session repetitions to failure
Kulik et al. [42]	Back squat	Failure × 5 × 85% 1RM, 3-min inter-set	CHO: 29.7 ± 3.6 PLA: 28.5 ± 3.0	Total session repetitions, sets, work, duration, and volume load
Lambert et al. [39]	Leg extension	Failure × 7–10 × 80% 10RM, 3-min inter-set	Unclear	Total session repetitions and sets
Laurenson et al. [32]	Back squat, bench press	6 × 7–15 × 60% 1RM, 2–5-min inter-set	Unclear	Volume and power during last set of each exercise
Naharudin et al. [75]	Back squat, bench press	4 × failure × 90% 10RM, 3-min inter-set	Unclear	Total repetitions completed per set and exercise
Oliver et al. [46]	Smith machine back squat	5 × 10 × 75% 1RM, 3-min inter-set	Unclear	Average power, velocity, and force per set and session
Rountree et al. [69]	Wall throw, sumo deadlift high pull, push press	5 × repetitions per min × 9–-34 kg, 1-min inter-set	30	Total repetitions completed per set and session
Smith et al. [41]	Mixture of upper body strength/hypertrophy exercises	5 × failure × 65% 1RM, 2-min inter-set	59.8 ± 2.3	Total repetitions completed per exercise and session
Wax et al. [44, 45]	Static quadriceps isometric contraction	Failure × 20 s × 50% MVF; 4–7 × 3 s × 100% MVC + ES, 40-s inter-set	CHO = 29 ± 13.1 PLA = 16.0 ± 8.1	Time to exhaustion Total force during 50%MVC and 100%MVC + ES
Wilburn et al. [43]	Leg press	4 × failure × 70% 1RM, 45-s inter-set	Unclear	Total repetitions completed per set and session

Values are expressed as means ± standard deviations

*BM* body mass, *CHO* carbohydrate, *CMJ* counter movement jump, *CON* control, *ES* electrostimulation, *F* female, *IRM* 1-repetition maximum, *10RM* 10-repetition maximum, *M* male, *MVC* maximum voluntary contraction, *PLA* placebo, *RT* resistance training

#### Nutrition Protocol

Of the 21 studies included in this review, 19 delivered CHO as a liquid beverage, one used a viscous semi-solid meal [75], and one used CHO-containing food items served as a meal [74]. All studies that delivered CHO as a liquid beverage or semi-solid meal used a simple, powdered CHO source such as maltodextrin, dextrose/glucose, or fructose. For the comparator condition, 18 studies reported using a low/non-caloric placebo; however, two studies did not explicitly provide information regarding the caloric content of the placebo beverage [33, 72]. One study used a water-only control condition [74] and one study used both a placebo and water-only control as comparator conditions [75]. There was a range of pre-trial fasting durations from 2 to12 hours. These pre-trial fasting durations clustered at each end of the range with nine studies using a 2- to 4- or 10- to 12-h fast duration, respectively. The fast duration of two studies was unclear [70, 72]. A comprehensive description of nutrition protocols of all studies is shown in Table 3.

**Table 3:** Nutrition protocol characteristics of the studies included in the systematic review

Study (year)	Pre-trial diet	Pre-trial fast (h)	CHO protocol (dose unit in g/kg body mass unless stated)		Placebo/control description
			CHO dose	Timing around training session	
Aoki et al. [33]	24-h prescribed diet (70% CHO, 15 fats, 15% protein)	2	60 g	1-h before (30 g) and ~ 10 min before (30 g)	AS beverage
Ballard et al. [71]	24-h prescribed diet (65% CHO, 20% fat, 15% protein)	2.5	65 g	5-min before and during (32 servings total)	AS non-caloric beverage
Battazza et al. [72]	24-h record of normal dietary habits	Unclear	60 g	1-h before	Unclear
Bin Naharudin et al. [74]	24-h record of normal dietary habits	10	1.5	2-h before	*Ad-libitum* water only
Bird et al. [73]	3-day record of normal dietary habits (~ 3.8 g/kg/day CHO)	4	25.2 g	15-min before (5.5 g) and after each set (19.7 g)	AS non-caloric beverage
dos Santos et al. [70]	None	Unclear	20 g	1-h before	Non-caloric beverage
Fairchild et al. [36]	24-h record of normal dietary habits	12	75 g	Immediately after first set of exercise	AS non-caloric beverage
Haff et al. [38]	3-day record with recommended diet (55% CHO, 20% protein, 25% fat)	2.5	0.3	After every second set to failure	AS non-caloric beverage
Haff et al. [34]	3-day record with recommended diet (55% CHO, 20% protein, 25% fat)	3	0.3–1.0	10-min before (1.0 g/kg) and every 10 min during (0.3 g/kg)	AS non-caloric beverage
Haff et al. [35]	3-day record with recommended diet (55% CHO, 20% protein, 25% fat)	3	0.51–1.0	Immediately before (1.0 g/kg) and after sets 1, 6, and 11 (0.51 g/kg)	AS non-caloric beverage
Krings et al. [40]	Instructed to maintain normal dietary habits	10	15, 30 and 60 g/h	Immediately before and every 15 min during	Low caloric amino acid-electrolyte beverage (~ 20 kcal)
Kulik et al. [42]	3-day record with recommended diet (55% CHO, 20% protein, 25% fat)	3	0.3	Immediately before and after every second set	AS non-caloric beverage
Lambert et al. [39]	2-day record of normal dietary habits	4	0.17–1.0	Immediately before (1.0 g/kg) and after set 5, 10, and 15 (0.17 g/kg)	Non-caloric beverage
Laurenson et al. [32]	3-day record of normal dietary habits	8	36 g	At 12 and 26 min during (18 g)	AS non-caloric beverage
Naharudin et al. et al. [75]	2-day record of normal dietary habits	10–13	1.5	2-h before	PLA: Semi-solid, low caloric CON: *Ad-libitum* water only
Oliver et al. [46]	24-h record of normal dietary habits	12	1.2	2-h before	AS non-caloric beverage
Rountree et al. [69]	Instructed to maintain normal dietary habits 3-days prior	10–12	16 g	Immediately before and after every round	AS non-caloric beverage
Smith et al. [41]	24-h record of normal dietary habits	10	36 g	Immediately before and after the last set of each exercise	AS non-caloric beverage
Wax et al. [44, 45]	3-day record with recommended diet (55% CHO, 20% protein, 25% fat)	10	0.17–1.0	30-min before (1.0 g/kg) and every 6 min during (0.17 g/kg)	AS non-caloric beverage
Wilburn et al. [43]	2-day record of normal dietary habits	3	2.0	30-min before	AS non-caloric beverage

*AS* artificially sweetened, *CHO* carbohydrate, *CON* control*, PLA* placebo

### Risk of Bias Assessment

**Fig. 2:**
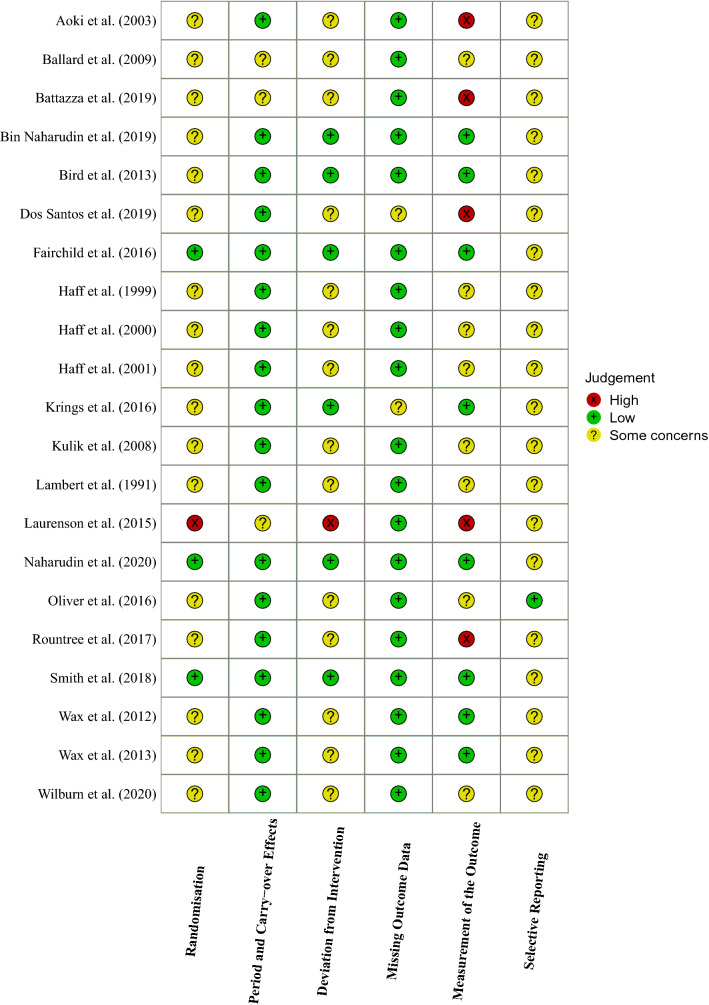
Risk of bias assessment for all included studies

One study was rated a high risk of bias related to the randomisation process [32]. Three studies [36, 41, 75] reported a randomisation method and information that would suggest allocation sequences were concealed (e.g., a researcher uninvolved in data collection handled randomisation and sequence allocation) and were awarded a low risk of bias. The remaining studies stated the trial was randomised but did not report a randomisation method or information regarding allocation concealment and were rated with some concerns. In the domain assessing risk of bias related to period and carry-over effects, three studies [32, 71, 72] did not report the wash-out length and were rated as having some concerns. The remaining studies reported a sufficient wash-out period (at least 72 h) and were rated as having a low risk of bias. Regarding bias arising from the intervention assignment, 14 studies were rated as having some concerns due to a lack of reported information as to whether participants and personnel delivering the intervention were aware of the intervention assigned. A high risk of bias was awarded to one study [32] due to a lack of blinding, whereas six studies presented information that suggested the participants and personnel delivering the intervention were not aware of the intervention assigned and were rated as having a low risk of bias [36, 40, 41, 73–75]. Relating to bias from missing outcome data, two studies were rated as having some concerns due to missing data points on presented figures [70] and due to having no provided reason for participant drop-outs that could have arisen due to the intervention [40]. The rest of the studies were rated as having a low risk of bias. Relating to risk of bias in the measurement of the outcome, five studies [32, 33, 69, 70, 72] were rated as having a high risk of bias for a lack of information on outcome assessor blinding, diet standardisation, or time of testing. Eight studies were rated as having some concerns for a lack of information to indicate outcome assessor blinding [34, 35, 38, 39, 42, 43, 46, 71]. The remaining studies were awarded a low risk of bias for this domain, 4 of which provided sufficient information to indicate assessor blinding [36, 40, 41, 73, 75], one was not able to blind assessors due to trial context [74], and in two, there was insufficient information to judge assessor blinding, but the outcomes were not likely affected by blinding [44, 45]. Regarding bias related to the selection of the reported result, all studies reported results in agreement with what was outlined in their methods sections. One study [46] pre-registered the trial protocol with a publicly available register and was rated as having a low risk of bias. The rest of the studies were not pre-registered and were rated as having some concerns. Risk of bias assessment is illustrated in the traffic light format in Fig. 2.

### Total Session Training Volume

**Fig. 3:**
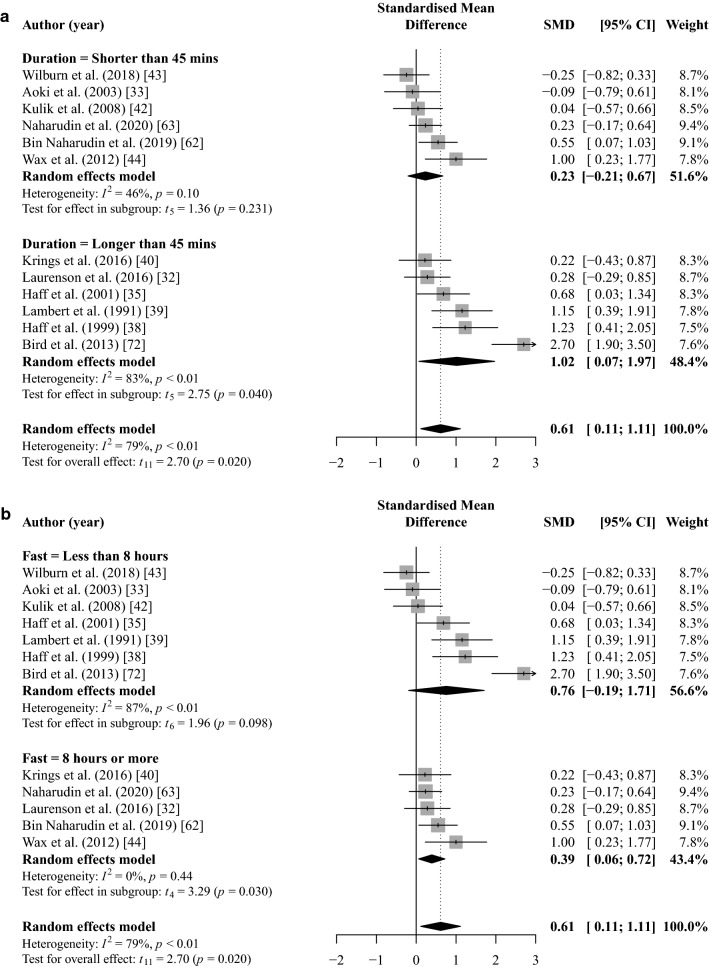
Random-effects meta-analysis of the effect of acute CHO ingestion on total training session volume compared to a placebo or water only. Sub-group analysis based on session (**a**) and fast (**b**) duration separately. *CHO* carbohydrate, *CI* confidence interval, *SMD* standardised mean difference

Pooled meta-analysis identified a significant benefit of CHO ingestion in comparison to a placebo or control for total session training volume (SMD = 0.61, [95% CI 0.11, 1.11]; *p* = 0.020; *I*^2^ = 79%; *k* = 12; Fig. 3). The meta-analysis for total session training volume provided low GRADE quality of evidence (Table 4). There was no evidence of publication bias for the training volume outcome (*b* = 5.26; [95% CI 0.21, 10.3]; t = 2.04; *p* = 0.069).

**Table 4:** Summary of meta-analysis findings and quality of evidence synthesis

Outcome	Summary of findings				Quality of evidence synthesis (GRADE)			
	k	n	Effect (95% CI)	Direction of the effect compared to placebo	Imprecision	Inconsistency	Risk of bias	Overall quality
**Total session volume**								
Longer than 45-min session duration	6	68	1.02 (0.07, 1.97)	↑	− 1	− 1	None	Low
Shorter than 45-min session duration	6	53	0.23 (− 0.21, 0.67)	↔	− 1	None	None	Moderate
8-h fast or more	5	61	0.39 (0.06, 0.72)	↑	− 1	None	None	Moderate
Less than 8-h fast	7	60	0.76 (− 0.19, 1.71)	↔	− 1	− 1	None	Low
All	12	121	0.61 (0.11, 1.11)	↑	− 1	− 1	None	Low
**Blood lactate**								
Higher than 45 min	4	36	0.50 (− 0.73, 1.74)	↔	− 1	− 1	None	Low
Lower than 45 min	3	22	0.66 (-0.18, 1.50)	↔	− 1	None	None	Moderate
All	7	58	0.58 (0.03, 1.14)	↑	− 1	− 1	None	Low
**Blood glucose**								
Longer than 45-min session duration	8	97	2.94 (1.67, 4.21)	↑	None	− 1	None	Moderate
Shorter than 45-min session duration	5	48	1.42 (− 1.55, 4.39)	↔	− 1	− 1	None	Low
8-h fast or more	4	46	1.58 (− 0.21, 3.38)	↔	− 1	− 1	None	Low
Less than 8-h fast	9	89	2.83 (1.09, 4.57)	↑	None	− 1	None	Moderate
All	13	135	2.36 (1.17, 3.55)	↑	None	− 1	None	Moderate

Only outcomes with *k* > 1 are included in this table

*CI* confidence intervals, *GRADE* Grading of Recommendations Assessment, Development and Evaluation, *k* number of studies, *n* number of participants

Sub-group analysis revealed a significant effect of CHO ingestion for session durations longer than 45 min (SMD = 1.02 [95% CI 0.07, 1.97]; *p* = 0.040; *I*^2^ = 83%; *k* = 6; Fig. 3). For session durations shorter than 45 min, CHO ingestion did not have a statistically significant effect on training volume (SMD = 0.23 [95% CI − 0.21, 0.67]; *p* = 0.231; *I*^2^ = 46%; *k* = 6; Fig. 3). These sub-group analyses provided low and moderate GRADE quality of evidence for longer and shorter than 45 min, respectively (Table 4).

Sub-group analysis revealed a significant effect of CHO ingestion for fasting periods ≥ 8 h (SMD = 0.39 [95% CI 0.06, 0.72]; *p* = 0.030; *I*^2^ = 0%; *k* = 5; Fig. 3). For fasting duration < 8 h, CHO ingestion did not have a significant effect on training volume (SMD = 0.76 [95% CI − 0.19, 1.71]; *p* = 0.09; *I*^2^ = 87%; *k* = 7; Fig. 3). These results provided moderate- and low-GRADE quality of evidence for fasting duration ≥ 8 h or < 8 h, respectively (Table 4).

**Fig. 4:**
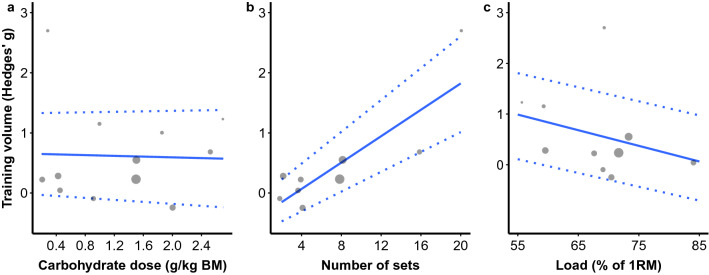
Mixed-effects meta-regression of the effect of acute CHO ingestion on RT volume performance compared to a placebo or water only while controlling for the effects of CHO dose (**a**), maximal effort sets completed (**b**), and load used (**c**). Larger data points received greater weighting than smaller data points. Solid lines represent the estimated relationship and dotted lines represent the upper and lower 95% confidence intervals. *BM* body mass, *CHO* carbohydrate, *IRM* 1-repetition maximum, *RT* resistance training

The total number of maximal effort sets (*b* = 0.11 [95% CI 0.05, 0.17]; *p* = 0.005) was a significant moderator of the SMD for training volume. CHO dose (*b* =  − 0.03 [95% CI − 0.68, 0.62]; *p* = 0.917) and load used (*b* =  − 0.03 [95% CI (− 0.11, 0.05); *p* = 0.400) were not significant moderators of the SMD for training volume (Fig. 4).

### Blood Lactate

**Fig. 5:**
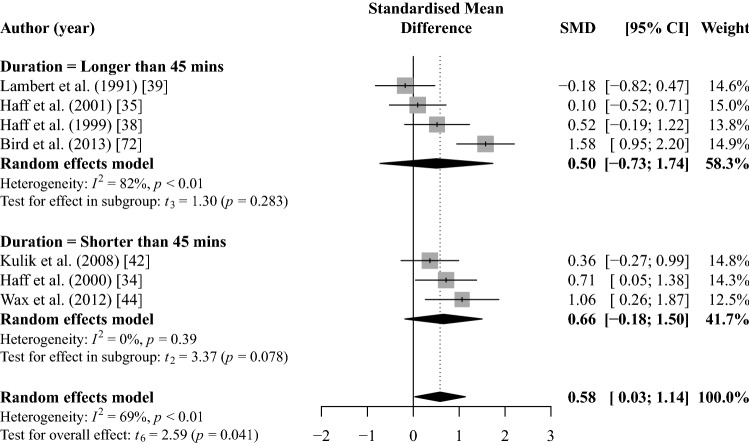
Random-effects meta-analysis of the effect of acute CHO ingestion on post-exercise blood lactate accumulation compared to a placebo or water only. Sub-group analysis based on session duration and post-exercise lactate *CHO* carbohydrate, *CI* confidence interval, *SMD* standardised mean difference

Pooled meta-analysis for post-exercise blood lactate identified significantly higher concentrations with CHO ingestion than a placebo or control (SMD = 0.58 [95% CI 0.03, 1.14]; *p* = 0.041; *I*^2^ = 69%; *k* = 7; Fig. 5) with a low-GRADE quality of evidence (Table 4).

Sub-group analysis indicated that post-exercise blood lactate concentrations were not significantly different for session duration ≥ 45 min (SMD = 0.50 [95% CI − 0.73, 1.74]; *p* = 0.283; *I*^2^ = 82%; *k* = 4; Fig. 5) or shorter than 45 min (SMD = 0.66 [95% CI − 0.18, 1.50]; *p* = 0.078 *I*^2^ = 0%; *k* = 3; Fig. 5). These results provided low and moderate GRADE quality of evidence, respectively (Table 4).

Carbohydrate dose was not a significant moderator of post-exercise blood lactate (*b* =  − 0.24 [95% CI − 0.93, 0.45]; *p* = 0.418). The total number of maximal effort sets, and load used were not meta-regressed for post-exercise blood lactate due to insufficient data.

### Blood Glucose

**Fig. 6:**
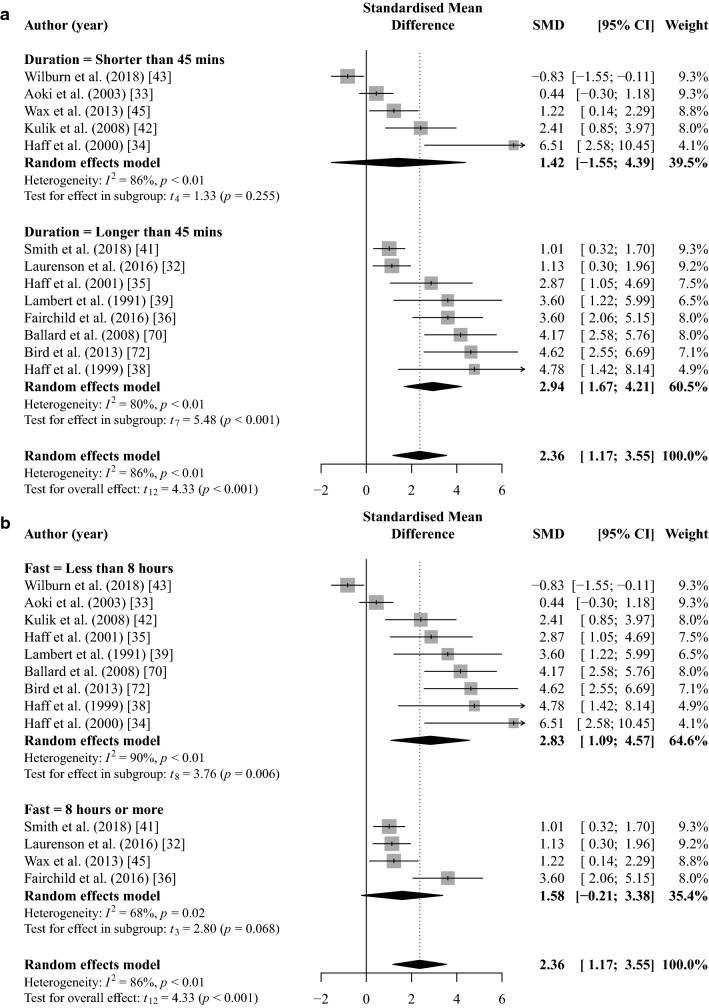
Random-effects meta-analysis of the effect of acute CHO ingestion on post-exercise blood glucose concentration compared to a placebo or water only. Sub-group analysis based on session (**a**) and fast (**b**) duration separately. *CHO* carbohydrate, *CI* confidence interval, *SMD* standardised mean difference

Pooled meta-analysis for post-exercise blood glucose identified significantly higher concentrations with CHO ingestion than a placebo or control (SMD = 2.36 [95% CI 1.17, 3.55]; *p* < 0.001; *I*^2^ = 86%; *k* = 13; Fig. 6) with a moderate GRADE quality of evidence (Table 4).

Sub-group analysis indicated that post-exercise blood glucose concentration was significantly higher for CHO ingestion in session duration ≥ 45 min (SMD = 2.94 [95% CI 1.67, 4.21]; *p* = 0.001; *I*^2^ = 80%; *k* = 8; Fig. 6). Post-exercise blood glucose concentration was not significantly different for session durations shorter than 45 min (SMD = 1.42 [95% CI − 1.55, 4.39]; *p* = 0.255; *I*^2^ = 86%; *k* = 5; Fig. 6). The session duration sub-group analysis provided moderate- and low-GRADE quality of evidence for longer and shorter than 45 min, respectively (Table 4).

Sub-group analysis indicated that post-exercise blood glucose concentration was not significantly higher for CHO ingestion following fasting duration of ≥ 8 h (SMD = 1.58 [95% CI − 0.021, 3.38]; *p* = 0.068; *I*^2^ = 68%; *k* = 4; Fig. 6), whereas post-exercise blood glucose was significantly higher for CHO ingestion following a fasting duration of < 8 h (SMD = 2.83 [95% CI 1.09, 4.57]; *p* = 0.006; *I*^2^ = 90%; *k* = 9; Fig. 6). The fasting duration sub-group analysis provided low and moderate GRADE quality of evidence for ≥ 8 h or < 8 h, respectively (Table 4).

**Fig. 7:**
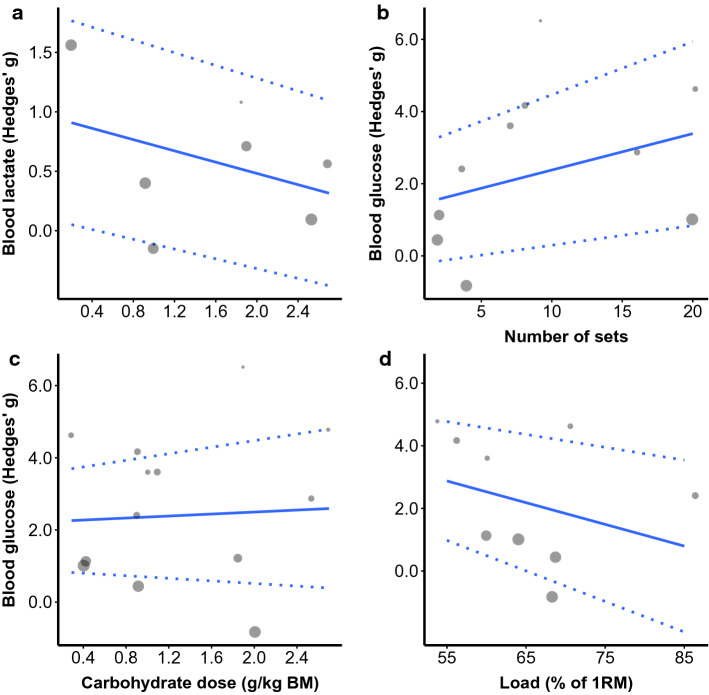
Mixed-effects meta-regression of the effect of acute CHO ingestion on post-exercise blood lactate and glucose compared to a placebo or water only while controlling for the effects of CHO dose on post-exercise lactate (**a**) and total number of maximal effort sets **(b)**, CHO dose (**c**), and load used (**d**) on post-exercise blood glucose. Larger data points received greater weighting than smaller data points. Solid lines represent the estimated relationship and dotted lines represent the upper and lower 95% confidence intervals. *BM* body mass, *CHO* carbohydrate, *CI* confidence interval, *IRM* 1-repetition maximum, *SMD* standardised mean difference

Carbohydrate dose (*b* = 0.14 [95% CI − 1.54, 1.82]; *p* = 0.859), number of maximal effort sets (*b* = 0.10 [95% CI − 0.12, 0.32]; *p* = 0.319), and load used (*b* =  − 0.07 [95% CI − 0.25, 0.11]; *p* = 0.400) were not significant moderators of post-exercise blood glucose concentration (Fig. 7).

### Sensitivity Analyses

Sensitivity analyses indicated that the pooled and subgroup training volume and post-exercise lactate meta-analyses were robust when imputing a within-study correlation of 0.3 and 0.5. Similarly, the post-exercise blood glucose meta-analyses for longer and shorter session duration and fasting subgroups were robust when imputing a within-study correlation of 0.5 and 0.7. For the 8 h or more fast sub-group, imputing with a 0.5 within-study correlation provided robust results (*p* > 0.05), but when imputing a correlation of 0.7, the result changed from non-significant (SMD = 1.58 [95% CI − 0.21, 3.38]; *p* = 0.068; *I*^2^ = 68%; *k* = 4) to significant (SMD = 1.51 [95% CI 0.14, 2.89]; *p* = 0.039; *I*^2^ = 77%; *k* = 4). A comprehensive report of the sensitivity analyses is provided in Supplementary Information Appendix S3.

## Discussion

The present systematic review and meta-analysis is the first to synthesise the evidence regarding the efficacy of CHO ingestion on resistance training performance and metabolic markers while also assessing potentially relevant moderators such as session duration, fasting duration, CHO dose, number of maximal effort sets, and load used. The main findings indicate that (1) CHO ingestion allows for greater RT volume to be completed, (2) CHO ingestion is effective for session durations longer than 45 min and fasting durations at least 8 h or more, (3) CHO ingestion elevates post-exercise blood lactate and glucose in comparison to a placebo or control, (4) the number of maximal effort sets moderates the effect of CHO ingestion on RT volume performance and post-exercise blood lactate, but not blood glucose, and (5) the load used and CHO dose do not moderate the effect of CHO ingestion on RT volume performance, post-exercise blood lactate, or post-exercise blood glucose.

### Total Training Session Volume

The present meta-analysis indicates that CHO ingestion results in a moderate effect size (SMD = 0.61 [95% CI 0.11, 1.11]) volume enhancement compared to a placebo or control. Given the novelty of the present review in quantitatively evaluating feeding strategies for RT performance, direct comparisons of this treatment effect with other CHO interventions are difficult. However, similar magnitudes of effect were reported for the effect of CHO feeding on mean power during cycling (SMD = 0.40–0.46) [76], and time to exhaustion (SMD = 0.47) and time trial performance in endurance exercise modalities (SMD = 0.53) [77]. In contrast, other acute ergogenic aids, such as caffeine (SMD = 0.20) [78] and citrulline malate (SMD = 0.30) [79] supplementation, have comparatively smaller magnitudes of effect on maximal strength performance.

Statistical heterogeneity in the present meta-analysis was high (*I*^2^ = 79%), indicating considerable variability in the effect size estimates across studies. Several studies reported large effect sizes with CHO ingestion [35, 38, 39, 44, 73]. All five of these studies used a training protocol consisting of only lower body training [35, 38, 39, 44, 73]. Conversely, studies including exercises of the upper body only or a mixture of upper and lower body exercise completed to failure, reported a non-significant effect of CHO ingestion on training volume performance [32, 40, 75]. For example, Bird et al. [73] reported a comparatively large effect size to the rest of the studies in the meta-analysis, in which participants completed 20 sets of lower body RT to failure. Lower body training recruits more total muscle mass, producing more total work, and subsequently, results in greater metabolic fatigue compared to upper body training [80–82]. However, this explanation is speculative, and may not be a lower body specific effect per se given that none of the studies included in this review included more than four sets of maximal effort upper body RT. Therefore, it is possible that if higher volumes of upper body RT are completed, CHO ingestion may also enhance volume for upper body RT similar to lower body RT. There are also several exceptions where volume was not enhanced for lower body RT. Specifically, Aoki et al. [33], Kulik et al. [42], and Wilburn et al. [43] all reported no improvement in total repetitions to failure during two to four sets of lower body RT. Therefore, it is likely that two to four sets of lower body exercise to failure was insufficient total volume to observe an ergogenic effect of CHO ingestion. This contention is supported by the results of our meta-regression analysis showing that the total number of sets performed with maximal effort is a significant moderator of the magnitude of ergogenic effects of CHO ingestion on RT performance. Overall, while CHO ingestion does have an ergogenic effect on RT performance, the magnitude of this effect is sensitive to the total amount of volume completed, with greater RT volumes (e.g., > 4 sets) benefitting more from CHO ingestion than lower RT volumes (e.g., ≤ 4 sets), and possibly lower body exercise selection (Fig. 4).

The results of our sub-group analyses indicate that session duration is important when considering the ergogenic effect of CHO, as volume was enhanced for session durations longer than 45 min (SMD = 1.02 [95% CI 0.07, 1.97]), but not shorter. There was high statistical heterogeneity observed among the studies in these subgroups, indicating substantial variability in the results. Again, the discrepancies in findings can likely be attributed to differences in the RT protocols and are highlighted by our meta-regression, which found that total sets completed with maximal effort (which directly influences session duration) is a significant moderator of the ergogenic effect of CHO on volume performance. Indeed, the decreases in muscle glycogen stores during RT are dependent on total training volume [15]. Unfortunately, muscle glycogen was only directly measured by one study included in the quantitative synthesis [43]. However, it could be hypothesised that without CHO ingestion, decreases in muscle glycogen stores influence fatigue in a time- (and volume-) dependant manner when the session duration exceeds 45 min, potentially constraining RT performance. Additionally, it could be hypothesised that CHO ingestion immediately before and during RT could supply blood glucose to the working musculature, and due to the intermittent nature of RT, be taken up by muscle during rest periods to aid in the partial replenishment of muscle glycogen. However, these notions are speculative and future research is needed to substantiate them. Nevertheless, the findings of this sub-group analysis indicate that CHO ingestion enhances training volume for RT sessions lasting greater than 45 min.

The fasting duration before RT is also an important consideration for the ergogenic effect of CHO ingestion, as the sub-group analysis indicates that CHO ingestion only enhances training volume after an 8-h or longer fast (SMD = 0.39 [95% CI 0.06, 0.72]). Extended periods of fasting inevitably lead to a decreased CHO availability, and exogeneous CHO may then be needed to ‘rescue’ performance. For instance, glycogen stores of the liver deplete during periods of fasting [21, 22], such as the overnight fast. The specialised glycogen stores of skeletal muscle are spared for high-intensity efforts and are thought to remain comparatively unaffected by periods of fasting [83]. However, acute feeding studies suggest that muscle glycogen stores can be partially depleted after an extended period of fasting, as muscle glycogen stores can increase 10–42% in the 3–4 h after a high CHO breakfast (approx. 2–3 g/kg body mass) [24, 25, 27], with a post-prandial period of at least 1–2 h necessary to achieve net gain in muscle glycogen stores [26]. The two studies with the largest effects for CHO ingestion in the ≥ 8-h fast subgroup provided CHO dose of 1.0–1.5 g/kg in the 0.5–2 h before RT [44, 74]; whereas, no volume enhancement was reported when CHO was ingested immediately before and during RT [32, 40]. Therefore, CHO ingestion in the hours before RT may be of importance for augmenting muscle glycogen stores and enhancing RT performance. In comparison to this finding, CHO ingestion after a fast of < 8 h did not enhance volume performance. In this sub-group, a small, CHO-containing breakfast was ingested 3–4 h before RT, which in addition to a moderate dietary CHO intake, was likely sufficient to preserve performance. It is also worth noting that there was high statistical heterogeneity in the results of the < 8-h fast sub-group (*I*^2^ = 87%), which again could be attributed to differences in the RT protocol (i.e., higher training volumes and lower body exercises). Overall, the findings of this sub-group analysis indicate that CHO ingestion attenuates the negative effect of extended fasting periods (≥ 8 h) on CHO availability, enhancing RT volume performance when compared to a control or placebo.

Our findings contrast and agree with the findings of a recent systematic review by Henselmans et al. [47], which found that the majority of studies assessing the effects of acute CHO ingestion on RT performance reported no ergogenic effect. There are differences in study inclusion criteria and outcomes of interest that may explain the differences in our findings. Specifically, the current review exclusively included cross-over trials comparing CHO ingestion to a zero-to-low kcal (≤ 25 total kilocalories) placebo or water-only control; whereas, the review by Henselmans et al. [47] additionally included parallel trials, and isocaloric comparator conditions. It is important to note that the analysis by Henselmans et al. [47] broadly and qualitatively assessed the effects of CHO ingestion on various RT outcomes, whereas we have used meta-analysis to specifically quantify the magnitude of the effects of CHO on RT volume performance. We have also conducted various sub-group and meta-regression analyses to control for potential confounders. Nonetheless, there is some agreement in results, as Henselmans et al. [47] note that CHO ingestion may be beneficial in some circumstances such as fasted training and higher training volumes (< 10 sets per muscle group). This finding by Henselmans et al. [47] agrees with the findings of our current meta-analysis in which CHO ingestion improves RT volume performance for longer session durations (> 45 min) and fast durations (≥ 8 h).

### Blood Lactate

Carbohydrate ingestion results in significantly higher post-exercise blood lactate accumulation (due to the greater work completed) in comparison to a placebo, with a moderate effect size (SMD = 0.58 [95% CI 0.03, 1.14]). Additionally, the duration of RT did not significantly affect post-exercise lactate accumulation. These findings are consistent with previous evidence that demonstrated less lactate accumulation with acute dietary CHO restriction during high-intensity exercise, when compared to a high dietary CHO intake [84]. Lactate is an important CHO fuel source during high-intensity exercise, and while lactate accumulation in blood is unlikely to be a central cause of fatigue during RT [85], post-exercise blood lactate is strongly correlated with metabolic and neuromuscular fatigue during high-intensity exercise [86, 87] and serves as a useful marker for fatigue evaluation. In the present meta-analysis, several studies reported significant increases in post-exercise blood lactate accumulation and reported large effect sizes for volume enhancement [44, 73]. On the other hand, Kulik et al. [42] reported similar post-exercise lactate between conditions and no volume enhancement. These findings suggest that the increased post-exercise lactate accumulation with CHO ingestion does not constrain RT performance and may even be necessary for improved performance. However, increased accumulation of post-exercise lactate with CHO ingestion suggests that total fatigue incurred from RT may increase due to the additional training volume performed. Therefore, a trade-off may exist where the cost of the ergogenic effect of CHO ingestion on RT volume induces additional metabolic stress and could influence time-course of recovery.

### Blood Glucose

Carbohydrate ingestion increases post-exercise blood glucose concentration with a large effect size (SMD = 2.36 [95% CI 1.17, 3.55]). In addition, CHO ingestion significantly increased post-exercise blood glucose for fasting durations less than 8 h (SMD = 2.83 [95% CI 1.09, 4.57]) and session durations longer than 45 min (SMD = 2.94 [95% CI 1.67, 4.21]). There was high heterogeneity across the post-exercise glucose findings, which could potentially be explained by the differences in participant cohorts amongst studies (e.g., training status, sex) (Table 1) and CHO dosages and timings (Table 3). There was consistently higher post-exercise glucose in studies that supplemented a rapidly digestible liquid CHO source during RT [34, 35, 38, 39, 42, 71, 73], whereas studies providing CHO in the 10–60 min before RT reported no increase in post-exercise blood glucose with CHO ingestion [33, 43]. These findings suggest that CHO ingestion increases blood glucose during RT and to maximise blood glucose availability, CHO ingestion should occur consistently during the RT session.

Several of the studies finding increased post-exercise blood glucose with CHO ingestion also reported improved training volume performance [35, 38, 39, 73]. However, it is presently unclear whether readily available blood glucose is necessary to improve RT performance under specific circumstances. A hypoglycaemic effect of RT training was not reported in any of the placebo conditions of the studies included in this review and blood glucose is maintained or increased after standard volumes of RT [15, 88]. Therefore, if blood glucose were to play a role in RT performance, it would likely be a result of maintaining or elevating blood glucose concentration as a readily available substrate for glycolysis or to partially replenish muscle glycogen during inter-set rest [28, 89]. Haff et al. [34] observed a significantly smaller decrease in muscle glycogen stores compared to resting values after RT with CHO ingestion (27%) when compared to a placebo (40%). Given that muscle glycogen stores are preferentially used to fuel specific processes during contraction, it is conceivable that at least some of this glycogen-sparing effect of CHO ingestion was a result of glycogenesis. Nevertheless, since it is presently unclear whether readily available blood glucose is necessary to improve RT performance, future studies should elucidate the effects of blood glucose on RT performance by manipulating pre-exercise CHO status and supplementing CHO during RT.

### Limitations and Considerations

There are several limitations to the current systematic review and meta-analysis that should be acknowledged. We opted to include only peer-reviewed, published literature in our review; the exclusion of grey literature could have biased the findings [90]. However, we note that funnel plot asymmetry examination and the results of the Egger’s regression test did not find publication bias to be present in the current review. While we contacted authors to request the data necessary for the analysis (e.g., correlations necessary for the calculation of the effect size variance), we were unable to acquire it. Therefore, we imputed correlations using unpublished data from our laboratory in our meta-analyses for all outcomes of interest. While this is a limitation to the current meta-analysis, sensitivity analyses with a range of other realistic correlations indicated that our results were largely robust to correlation imputations. Additionally, the data for two studies [41, 71] were originally intended to be used in the quantitative synthesis, but due to the data reporting and because we were unable to obtain the data from the authors before the analysis, they were ultimately omitted from the meta-analysis. Both investigations included upper body RT exercise completed to failure and could have contributed to an under-representation of upper body RT in the current meta-analysis. Finally, the GRADE quality of evidence presented in the current review was generally low to moderate. These ratings constrain the certainty of the results presented, but we have offered potential explanations for the heterogeneity and imprecision of the results to aid in interpretation. Additionally, given that CHO ingestion is unlikely to negatively affect performance and that the confidence limits of the present meta-analysis suggest at least a trivial ergogenic effect for volume enhancement, our overall recommendations reflect the position that CHO ingestion is an efficacious nutrition strategy for enhancing volume.

Several study characteristics warrant investigation in future research. Participants in the current review were generally consuming moderate amounts of dietary CHO in the 1–3 days preceding RT, and it is possible that varying amounts of dietary CHO could influence the ergogenic effect of acute CHO ingestion. Eight of 12 studies in the current meta-analysis used an exclusively lower body RT protocol; more research is needed to quantify the overall effect of CHO ingestion on upper body only RT, or a mixture of upper and lower body exercises. Regarding the generalisability of our results, only 12 of 226 (5.3%) participants included in this review were females. More research with female participants is therefore necessary to determine if sex-specific recommendations for CHO ingestion are needed, and what they should be. Additionally, we only identified one performance outcome that had sufficient data to enable meta-analysis. More research is needed on other outcomes such as expressions of muscle force production (e.g., maximal strength and power), muscle endurance, and time course of recovery to fully understand how CHO ingestion affects other RT performance indices. Finally, several recent investigations suggested that RT performance may be influenced by the psychological effects of, or the hunger and satiety cues associated with feeding [75, 91], a notion that is somewhat supported by our meta-regression finding that CHO dose was not a moderator of the ergogenic effect of CHO ingestion on RT performance. Future research should seek to fully elucidate the role of psychology and hunger/satiety on RT performance.

Several reporting and methodological issues were identified in the risk of bias analysis (Fig. 2). It was often unclear from the full texts what randomisation method was used, how allocation concealment was achieved, and how double blinding was achieved, and whether it was successful. An assessment of blinding efficacy may be informative in some circumstances, such as where participants can be blinded to their performance. We have not discussed blinding efficacy in the current review as participants are generally not able to be blinded to training volume completion. Moreover, the most recent Cochrane guidance notes that successful intervention guesses could simply reflect a good outcome of an active intervention (e.g., greater training volume performed could be attributed to CHO ingestion), and that deducing the intervention received does not inherently lead to a risk of bias (https://www.riskofbias.info/welcome/rob-2-0-tool/current-version-of-rob-2). Additionally, only one study included in the current review pre-registered their protocol and statistical reporting was often incomplete (e.g., missing means, standard deviation of the difference scores etc.). The quality of reporting seems to be improving with the recent publications being the only studies to report these methodological aspects in full, but this is not a consistent trend. To strengthen the quality of research on this topic, and to support open and transparent science, we encourage authors of future research to report methods and results in sufficient detail [92], readily provide study data to other researchers upon reasonable request, and to consider publicly pre-registering their investigations.

### Implications for Practice

The findings of the current review have several implications for practice:

1. For RT session durations greater than 45 min and consisting of at least 8–10 sets, CHO ingestion can be expected to improve performance.
2. When RT occurs after a ≥ 8-h fast, such as the overnight fast, CHO ingestion may improve performance relative to a control or placebo.
3. The number of sets completed with maximal effort seems to influence the ergogenic effect CHO ingestion. Therefore, as session training volume increases, the importance of CHO ingestion for performance also increases.
4. Carbohydrate ingestion seems to have a greater benefit for lower body RT protocols, suggesting that CHO ingestion before and during lower body RT sessions may be of importance.
5. Carbohydrate dose does not seem to influence the ergogenic effect of CHO ingestion. Therefore, ingesting an amount of CHO that the trainee perceives as adequate fuelling for the training session and to stave off sensations of hunger, may be of importance.
6. Carbohydrate ingestion enhances volume, which increases post-exercise blood lactate. While this increased lactate accumulation may be necessary for improved RT performance, there may be a trade-off where the additional fatigue incurred from greater training volume with CHO ingestion may influence the time-course of recovery.
7. Blood glucose may influence training volume as a readily available fuel source. To increase blood glucose during RT, it appears that readily digestible sources of CHO (e.g., a sports drink) during RT can consistently and robustly increase blood glucose concentration.

## Conclusions

This systematic review and meta-analysis found that the ingestion of CHO provides an ergogenic effect on RT volume performance, when compared to a placebo or control. Carbohydrate ingestion has ergogenic effects on RT performance where session duration was longer than 45 min and the fast duration was ≥ 8 h. Conversely, CHO ingestion did not significantly affect performance when session durations were shorter than 45 min or fast durations < 8 h. Post-exercise blood lactate is significantly higher with CHO ingestion compared to a placebo. Lactate itself is an important fuel source for training, but also strongly correlates with metabolic fatigue, suggesting that the additional lactate accumulation with CHO ingestion is necessary for RT performance, but the increased volume of training may incur additional fatigue. Post-exercise blood glucose was elevated with CHO ingestion, where readily digestible sources ingested during training seem to increase blood glucose the most. Meta-regression analysis revealed that sets completed with maximal effort was a significant moderator of the effect magnitude of CHO ingestion on RT performance and lactate, but not blood glucose. Load used and CHO dose were not significant moderators of the effect magnitude of CHO ingestion. Collectively, the findings of the current review demonstrate an ergogenic effect of CHO ingestion for enhancing volume performance during RT.

## Supplementary Information

Below is the link to the electronic supplementary material.

Supplementary file1 (DOCX 19 kb)

Supplementary file2 (DOCX 19 kb)

Supplementary file3 (DOCX 19 kb)

## Declarations

### Availability of data and material

The datasets and associated code for the analyses performed are available on the Open Science Framework (https://osf.io/hjfbw/).
