# Supplementary material for: The Ergogenic Effects of Acute Carbohydrate Feeding on Resistance Exercise Performance: A Systematic Review and Meta-analysis
Source: Sports Med. 2022 Jul 9;52(11):2691–712. doi: 10.1007/s40279-022-01716-w (PMC9584980; doi:10.1007/s40279-022-01716-w)
Supplement: Supplementary file 1 — Supplementary file1 (DOCX 19 kb) [file 40279_2022_1716_MOESM1_ESM.docx]

**Title**

The Ergogenic Effects of Acute Carbohydrate Feeding on Resistance Exercise Performance: A Systematic Review and Meta-analysis

**Journal**

Sports Medicine

**Authors**

Andrew King^1^, Eric Helms^1^, Caryn Zinn^1^, and Ivan Jukic^1^

**Affiliations**

^1^Sport Performance Research Institute New Zealand (SPRINZ), Auckland University of Technology, Auckland, New Zealand

**Corresponding author**

Andrew King

MSc Candidate

Sport Performance Research Institute New Zealand (SPRINZ)

Auckland University of Technology

17 Antares Place, Mairangi Bay

Auckland, New Zealand, 0632

Email: andrewking.biz@gmail.com

**Supplementary File I**

**Search Strings:**

**Pubmed:**

("carbohydrate" OR "glucose" OR "maltodextrin") AND ("resistance training" OR "weight training" OR "strength training" OR "resistance exercise")

**EBSCOhost (SportDiscus + CINAHL + MEDLINE):**

("carbohydrate" OR "glucose" OR "maltodextrin") TI TITLE

AND ("resistance training" OR "weight training" OR "strength training" OR "resistance exercise") TX All Text

**Scopus:**

TITLE ("carbohydrate" OR "glucose" OR "maltodextrin") AND TITLE-ABS-KEY ("resistance training" OR "weight training" OR "strength training" OR "resistance exercise")
