# Supplementary material for: The Ergogenic Effects of Acute Carbohydrate Feeding on Resistance Exercise Performance: A Systematic Review and Meta-analysis
Source: Sports Med. 2022 Jul 9;52(11):2691–712. doi: 10.1007/s40279-022-01716-w (PMC9584980; doi:10.1007/s40279-022-01716-w)
Supplement: Supplementary file 2 — Supplementary file2 (DOCX 19 kb) [file 40279_2022_1716_MOESM2_ESM.docx]

**Title**

The Ergogenic Effects of Acute Carbohydrate Feeding on Resistance Exercise Performance: A Systematic Review and Meta-analysis

**Journal**

Sports Medicine

**Authors**

Andrew King^1^, Eric Helms^1^, Caryn Zinn^1^, and Ivan Jukic^1^

**Affiliations**

^1^Sport Performance Research Institute New Zealand (SPRINZ), Auckland University of Technology, Auckland, New Zealand

**Corresponding author**

Andrew King

MSc Candidate

Sport Performance Research Institute New Zealand (SPRINZ)

Auckland University of Technology

17 Antares Place, Mairangi Bay

Auckland, New Zealand, 0632

Email: andrewking.biz@gmail.com

**Supplementary File II**

**Statistical Analysis: Additional information**

1. **Decisions on Total Session Volume**

Two studies [35, 44] used a protocol other than traditional RT but reported outcomes that were representative of the ability to complete volume across the RT session, and these outcomes were included in the meta-analysis. Specifically, Wax et al. [44] reported total session force output during submaximal isometric contraction of the quadriceps and Haff et al. [35] reported total session work across 16 sets of isokinetic knee extension/flexion. Furthermore, sub-group analyses for pre-trial fasting duration and total training session duration were performed for each of these three outcomes.

1. **Composite Effect Calculations**

Four studies [32, 33, 74, 75] reported training volume per exercise or set (e.g., total repetitions completed in the back squat and bench press), and these observations were combined into one effect per study. The study by Krings et al. [40] included four trials: a placebo and three different CHO dosages, of which the three CHO trials were combined into one effect for meta-analysis. The study by Naharudin et al. [75] compared a CHO trial to a placebo and water only control; thus, the observations of the placebo and control trials were combined into a composite effect. Two studies [41, 71] were originally intended to be used in the quantitative synthesis, but due to the data reporting and because we were unable to obtain the data from the authors before the analysis, they were ultimately omitted from the meta-analysis.

**(c) Meta-regression Calculations/Rationale**

The included studies reported various units for CHO dose (e.g., g/hr, g/kg, or total g) and load (e.g., 80% of a 10-RM). To obtain a CHO dose in g/kg body mass, estimations were made from information reported in the studies (e.g., a total CHO gram dosage was divided by the reported mean body mass of participants to obtain g/kg body mass). Similarly, an estimation for a %1-RM load was made if the prescribed load was not reported relative to a 1-RM. For total number of maximal effort sets, only repetitions or sets that were performed to failure or isokinetic repetitions that were completed with maximal effort were used. Three studies [38, 39, 44] were omitted from the total maximal effort sets meta-regression because the RT protocol resulted in total sets not being equated between CHO and placebo conditions.

**(d) Decisions on Publication Bias**

Publication bias for post-exercise blood glucose was not reported since blood glucose concentration was not a primary outcome for any individual study included in this review. More importantly, there was no reason to suspect that the presence or absence of a statistically significant for post-exercise blood glucose between CHO ingestion and placebo conditions would affect the chances of a study being published. Additionally, individual studies in the present review generally had small sample sizes (which were similar across the studies), with vastly different methodologies.
