# Supplementary material for: The Ergogenic Effects of Acute Carbohydrate Feeding on Resistance Exercise Performance: A Systematic Review and Meta-analysis
Source: Sports Med. 2022 Jul 9;52(11):2691–712. doi: 10.1007/s40279-022-01716-w (PMC9584980; doi:10.1007/s40279-022-01716-w)
Supplement: Supplementary file 3 — Supplementary file3 (DOCX 19 kb) [file 40279_2022_1716_MOESM3_ESM.docx]

**Title**

The Ergogenic Effects of Acute Carbohydrate Feeding on Resistance Exercise Performance: A Systematic Review and Meta-analysis

**Journal**

Sports Medicine

**Authors**

Andrew King^1^, Eric Helms^1^, Caryn Zinn^1^, and Ivan Jukic^1^

**Affiliations**

^1^Sport Performance Research Institute New Zealand (SPRINZ), Auckland University of Technology, Auckland, New Zealand

**Corresponding author**

Andrew King

MSc Candidate

Sport Performance Research Institute New Zealand (SPRINZ)

Auckland University of Technology

17 Antares Place, Mairangi Bay

Auckland, New Zealand, 0632

Email: andrewking.biz@gmail.com

**Supplementary File III**

**Total Training Volume**

**Pooled Meta-analysis**

Sensitivity analysis indicated that this effect was robust when imputing a within-study correlation of r = 0.3 (SMD = 0.53 [95% CI: 0.07, 1.00]; p = 0.027; *I*^2^ = 64%; k = 12) and r = 0.5 (SMD = 0.58 [95% CI: 0.10, 1.06]; p = 0.023; *I*^2^ = 69%; k = 12).

**Session Duration**

For longer than 45 mins, sensitivity analysis indicated that the significant effect was robust when imputing a within-study correlation of r = 0.3 (SMD = 0.97 [95% CI: 0.04, 1.90]; p = 0.043; *I*^2^ = 70%; k = 6) and r = 0.5 (SMD = 1.00 [95% CI: 0.06, 1.95]; p = 0.041; *I*^2^ = 75%; k = 6).

For short than 45 mins, sensitivity analysis indicated that the non-significant result was robust when imputing a within-study correlation of r = 0.3 (SMD = 0.21 [95% CI: -0.21, 0.63]; p = 0.253; *I*^2^ = 37%; k = 6) and r = 0.5 (SMD = 0.22 [95% CI: -0.21, 0.65]; p = 0.246; *I*^2^ = 41%; k = 6).

**Fast Duration**

For 8 hours or longer, sensitivity analysis indicated that the effect was robust when imputing a within-study correlation of r = 0.3 (SMD = 0.37 [95% CI: 0.06, 0.69]; p = 0.030; *I*^2^ = 0%; k = 5) and r = 0.5 (SMD = 0.38 [95% CI: 0.06, 0.71]; p = 0.031; *I*^2^ = 0%; k = 5)

For less than 8 hours, sensitivity analysis indicated that the non-significant result was robust when imputing a within-study correlation of r = 0.3 (SMD = 0.72 [95% CI: -0.22, 1.66]; p = 0.111; *I*^2^ = 78%; k = 7) and r = 0.5 (SMD = 0.75 [95% CI: -0.20, 1.70]; p = 0.103; *I*^2^ = 82%; k = 7).

**Meta-regressions**

The results were robust for an imputed correlation of 0.5:

CHO dose (b = 0.02 [95% CI: -0.62, 0.66]; p = 0.943)

Sets (b = 0.09 [95% CI: 0.03, 0.16]; p = 0.012)

Load used (b = -0.03 [95% CI: -0.11, 0.04]; p = 0.337)

The results were robust for an imputed correlation of 0.3:

CHO dose (b = 0.04 [95% CI: -0.58, 0.66]; p = 0.879)

Sets (b = 0.08 [95% CI: 0.02, 0.15]; p = 0.002)

Load used (b = -0.03 [95% CI: -0.11, 0.04]; p = 0.322)

**Blood Lactate**

**Pooled**

Sensitivity analysis indicated that this effect was robust when imputing a within-study correlation of r = 0.3 (SMD = 0.55, [95% CI: 0.01, 1.09]; p = 0.047; *I*^2^ = 57%; k = 7) and r = 0.5 (SMD = 0.57, [95% CI: 0.02, 1.11]; p = 0.044; *I*^2^ = 62%; k = 7).

**Session Duration**

For longer than 45 mins, sensitivity analysis indicated that the effect was robust when imputing a within-study correlation of r = 0.3 (SMD = 0.48; 95%CI [-0.72, 1.68]; p = 0.295; *I*^2^ = 75%; k = 4) and 0.5 (SMD = 0.49; 95%CI [-0.72, 1.71]; p = 0.288; *I*^2^ = 78%; k = 4)

For shorter than 45 mins, sensitivity analysis indicated that the effect was robust when imputing a within-study correlation of r = 0.3 (SMD = 0.64; 95%CI [-0.20, 1.48]; p = 0.082; *I*^2^ = 0%; k = 3) and 0.5 (SMD = 0.65; 95%CI [-0.20, 1.49]; p = 0.080; *I*^2^ = 0%; k = 3)

**Meta-regressions**

The results were robust for an imputed correlation of 0.5:

Dose (b = -0.22 [95% CI: -0.91, 0.48]; p = 0.460)

The results were robust for an imputed correlation of 0.3:

Dose (b = -0.20 [95% CI: -0.90, 0.49]; p = 0.489)

**Blood Glucose**

**Pooled**

Sensitivity analysis indicated that this effect was robust when imputing a within-study correlation of r = 0.5 (SMD = 2.38, [95% CI: 1.23, 3.52]; p < 0.001; *I*^2^ = 89%; k = 13) and r = 0.7 (SMD = 2.33, [95% CI: 1.25, 3.41]; p < 0.001; *I*^2^ = 92%; k = 14).

**Session Duration**

For longer than 45 mins, sensitivity analysis indicated the results were robust when imputing a within-study correlation of r = 0.5 (SMD = 2.88 [1.70, 4.06]; p < 0.001; *I*^2^ = 84%; k = 8) and r = 0.7 (SMD = 2.73 [1.66, 3.79]; p < 0.001; *I*^2^ = 87%; k = 8).

For shorter than 45 mins, sensitivity analysis indicated the results were robust when imputing a within-study correlation of r = 0.5 (SMD = 1.60 [-1.48, 4.68]; p = 0.223; *I*^2^ = 89%; k = 5) and r = 0.7 (SMD = 1.73 [-1.37, 4.82]; p = 0.196; *I*^2^ = 92%; k = 5).

**Fast Duration**

For 8 hours or more, sensitivity analysis indicated the results were robust when imputing a within-study correlation of r = 0.5 (SMD = 1.58; 95%CI [-0.08, 3.23]; p = 0.056; *I*^2^ = 74%; k = 4) but were not robust when imputing with r = 0.7 (SMD = 1.51; 95%CI [0.14, 2.89]; p = 0.039; *I*^2^ = 77%; k = 4).

For less than 8 hrs, sensitivity analysis indicated the results were robust when imputing a within-study correlation of r = 0.5 (SMD = 2.83; 95%CI [1.17, 4.50]; p = 0.004; *I*^2^ = 92%; k = 9) and r = 0.7 (SMD = 2.76; 95%CI [1.20, 4.33]; p = 0.004; *I*^2^ = 94%; k = 9).

**Meta-regression**

The results were robust for an imputed correlation of 0.5:

Dose (b = 0.24 [95% CI: -0.14, 1.84]; p = 0.753)

Sets (b = 0.10 [95% CI: -0.12, 0.31]; p = 0.330)

Load used (b = -0.07 [95% CI: -0.24, 0.11]; p = 0.392)

The results were robust for an imputed correlation of 0.7:

Dose (b = 0.35 [95% CI: -0.14, 1.84]; p = 0.615)

Sets (b = 0.09 [95% CI: -0.12, 0.29]; p = 0.346)

Load used (b = -0.06 [95% CI: -0.22, 0.10]; p = 0.389)
